# Impact of Long COVID on Health-Related Quality of Life Among COVID-19 Survivors in Saudi Arabia

**DOI:** 10.3390/healthcare13080890

**Published:** 2025-04-12

**Authors:** Mohammed A. BuSaad, Adam F. Aldhawyan, Batool A. Alattas, Rahaf S. AlAlloush, Mohammed A. Alharbi, Nourah K. Alkaltham, Assim AlAbdulKader, Reem S. AlOmar

**Affiliations:** 1 Department of Family and Community Medicine, College of Medicine, Imam Abdulrahman Bin Faisal University, Dammam 34221-4237, Saudi Arabia; aaaldhawyan@iau.edu.sa (A.F.A.); nkalkaltham@iau.edu.sa (N.K.A.); aabdulkader@iau.edu.sa (A.A.); rsomar@iau.edu.sa (R.S.A.); 2 College of Medicine, Imam Abdulrahman Bin Faisal University, Dammam 34221-4237, Saudi Arabia; 71batool@gmail.com (B.A.A.); rahafalalloush@gmail.com (R.S.A.); m.alhumaidi.alharbi@gmail.com (M.A.A.)

**Keywords:** long COVID, epidemiology, public health, HRQoL, EQ-5D-5L, COVID-19, quality of life, Saudi Arabia

## Abstract

**Background**: Long COVID (LC) has emerged as a significant epidemiological and public health issue, affecting patients’ health-related quality of life (HRQoL). This study explored the impact of LC on HRQoL in COVID-19 survivors in the Eastern Province of Saudi Arabia and examined the sociodemographic and clinical factors that influence HRQoL. **Methods**: This cross-sectional study included 1024 participants, and data were collected through face-to-face interviews using a structured questionnaire that incorporated the EQ-5D-5L tool to assess HRQoL. Sociodemographic information, acute COVID-19 symptoms, and LC symptoms were recorded. Statistical analyses included bivariate analyses and multivariable generalized linear modelling. **Results**: Of all participants, 63.8% reported experiencing LC symptoms, with fatigue, cough, and anosmia being the most common. Participants with LC had significantly lower HRQoL scores (mean EQ-5D-5L index score, 0.93) than those without LC (mean score, 0.98; *p* < 0.001). The key factors influencing lower HRQoL included a higher number of LC symptoms, older age, the presence of pneumonia during acute COVID-19, and pre-existing conditions such as anxiety and hypertension. **Conclusions**: LC negatively impacts HRQoL, with older age, chronic diseases, and the number of LC symptoms being strong predictors of poor outcomes. Interventions targeting rehabilitation and psychosocial support are critical for improving the long-term health outcomes of patients with LC.

## 1. Introduction

The Coronavirus disease 2019 (COVID-19) pandemic has significantly impacted societies worldwide, leading to widespread health, social, and economic challenges [1]. The COVID-19 pandemic has significantly shifted global health perspectives, affecting quality of life and well-being. The pandemic has underscored the critical role of the healthcare infrastructure, the need for mental health support, and the vulnerabilities of individuals with pre-existing conditions. Prolonged isolation has highlighted the importance of mental health, promoting a holistic approach that includes emotional and psychological well-being. Ultimately, COVID-19 emphasizes the importance of proactive measures to maintain overall health [2].

SARS-CoV-2, the virus responsible for COVID-19, shares approximately 82% genomic similarity with SARS-CoV-1, which emerged in 2002 [3]. It is an enveloped, positive-sense single-stranded RNA virus with a genome structured into three key regions: the 5′ terminal, a central section containing open reading frames essential for replication, and the 3′ terminal, which encodes five structural proteins—Spike (S), membrane (M), nucleocapsid (N), envelope (E), and hemagglutinin-esterase (HE) [3,4]. The Spike protein plays a central role in infectivity via its receptor-binding domain (RBD), which binds to the Angiotensin-Converting Enzyme 2 (ACE-2) receptor on human cells, facilitating entry through transmembrane protease serine 2 (TMPRSS2) expression [5].

The widespread distribution of ACE-2 across various organs allows SARS-CoV-2 to affect systems beyond the lungs, including the kidneys, heart, blood vessels, liver, pancreas, and immune system [6]. ACE-2 is also expressed in the digestive tract, testis, and spleen, providing additional pathways for viral entry. The virus triggers an exaggerated immune response, with excessive cytokine and chemokine production, leading to a cytokine storm that increases the risk of acute respiratory distress syndrome (ARDS) and multi-organ failure [7].

Despite growing recognition, little attention has been devoted to the management and prevention of long COVID (LC) and its impact on health-related quality of life (HRQoL). LC is defined as a chronic condition following SARS-CoV-2 infection that lasts at least three months and may be continuous, relapsing, or progressively worsening. It can affect multiple organ systems, and diagnosis does not require the laboratory confirmation of the initial infection [8].

The ongoing debate in the literature highlights the uncertainty surrounding the optimal management of LC. Due to the lack of reliable evidence-based guidelines, patients are often exposed to unproven treatments, some of which may be costly or potentially harmful. Even for interventions with published trials, such as physical rehabilitation and cognitive behavioural therapy, concerns about their credibility persist among both patients and healthcare providers. High-quality systematic reviews are essential to evaluate the benefits and risks of available treatments, ensuring evidence-based care for LC patients [9].

Multiple studies conducted in Switzerland, Belgium, Japan, and India have examined the impact of LC symptoms on HRQoL using the validated and widely used EuroQol 5-Dimensions 5-Level (EQ-5D-5L) questionnaire [10]. This tool assesses five key dimensions—mobility, self-care, usual activities, pain/discomfort, and anxiety/depression—across five levels of severity. A notable study in an Irish cohort of 988 individuals with symptoms persisting beyond 14 days found that 89% had not returned to their pre-COVID-19 health status, with a median time of 12 months since acute infection [11]. The majority were female (88%) and white (98%), with a median age of 43 years and a median body mass index (BMI) of 26.

Participants reported a median of eight ongoing symptoms. Notably, 38% indicated severe limitations in their ability to work, and 33% experienced moderate or higher levels of anxiety or depression. These findings align with the broader literature confirming that LC patients often experience persistent, multi-system symptoms that impair both quality of life and occupational function. The study underscores the importance of dedicated multidisciplinary care to improve outcomes for this population [11].

The EQ-5D-5L has also proven effective in capturing HRQoL changes across various conditions [12]. Its sensitivity has been shown in chronic heart failure, distinguishing symptom severity [13], and in diabetes, capturing utility differences across complications and treatments [14]. Its role in calculating quality-adjusted life years (QALYs) further supports its use in both clinical and economic evaluations [12,15].

Despite some criticism of its sensitivity to complex health states, particularly in mental health, the EQ-5D-5L remains central to HRQoL research and policymaking [10,16]. In previous COVID-19 studies, a significant proportion of recovered individuals reported lasting impacts on HRQoL. Fatigue was the most frequently reported symptom, followed by pain/discomfort, anxiety, depression, memory problems, and hair loss [17,18,19]. Other studies have also documented considerable variation in the prevalence and severity of LC symptoms, reflecting diverse perceptions of symptom burden among affected individuals [20,21].

Therefore, this study aimed to evaluate the impact of SARS-CoV-2 infection and LC HRQoL in the Eastern Province of Saudi Arabia, given their emergence as significant public health concerns. Additionally, it sought to identify the sociodemographic and clinical factors associated with the trajectory of HRQoL following infection.

## 2. Materials and Methods

### 2.1. Study Design and Participants

This cross-sectional study was conducted in the Eastern Province of Saudi Arabia from April to August 2024, after approval by the Institutional Review Board of Imam Abdulrahman Bin Faisal University (IRB-2024-01-208). Informed consent was obtained from all participants prior to completing the questionnaire.

With a precision of 5% and an alpha level of 0.05, the sample size was calculated using Epi Info 7.0, resulting in a minimum required sample size of 251. This study utilized a non-probability sampling approach to recruit participants. Inclusion criteria comprised adults aged 18 years and older residing in the Eastern Province of Saudi Arabia with a confirmed COVID-19 diagnosis by reverse transcription polymerase chain reaction (RT-PCR) testing. Exclusion criteria included refusal to provide informed consent or failure to disclose essential information required for the study.

### 2.2. Questionnaire Development and Data Collection

Data were collected by trained volunteers through face-to-face interviews using a tablet-based questionnaire in public community settings. The questionnaire consisted of two parts: the first part gathered information on sociodemographic and clinical characteristics, while the second part comprised the EQ-5D-5L, a standardized tool for assessing health-related quality of life (HRQoL).

The sociodemographic and clinical questionnaire was developed by the authors and included multiple items. It assessed key aspects such as sociodemographic characteristics, risk factors, the time of infection, acute symptoms, and persistent manifestations. This section aimed to provide a detailed profile of participants to better understand how their backgrounds might relate to their experiences with COVID-19. Two family physicians reviewed the questionnaire to ensure content accuracy and relevance.

The EQ-5D-5L questionnaire, developed by the EuroQol Group in 2009, was employed to evaluate health-related quality of life (HRQoL). This validated and widely used instrument is composed of two pages [22]. The first includes five dimensions—mobility, self-care, usual activities, pain/discomfort, and anxiety/depression—each rated on a 5-level Likert scale ranging from 1 (no problems) to 5 (extreme problems/unable to do so). The second page features the EuroQol Visual Analogue Scale (EQ-VAS), a vertical scale ranging from 0 to 100, where 0 represents ‘The worst health you can imagine’ and 100 represents ‘The best health you can imagine’. Using nation-specific value sets, an index value was calculated for each potential EQ-5D-5L health status.

### 2.3. Statistical Analysis

Continuous variables are described as the mean ± standard deviation (SD). The Kolmogorov–Smirnov test assessed the statistical normality assumption of the metric variables. Metric variables that violated the normality assumption, such as skewness, are described using the median and interquartile range (IQR). Categorical variables are described as frequencies and percentages, and multiple response dichotomies analysis was used for variables with multiple options, such as COVID-19 symptoms. The reliability of the measured questionnaires/scales was assessed using Cronbach’s alpha for internal consistency, yielding a value of 0.781, which indicates good internal consistency and supports the reliability of the scale. Bivariate Spearman’s correlation (rho) tested the correlations between metric variables. An independent samples *t*-test assessed the statistical significance of mean differences in metric variables across levels of binary dichotomous variables. In addition, multivariable generalized linear modelling with gamma regression, using the maximum likelihood method, was applied to an individual’s mean perceived HRQoL scale score by regressing the HRQoL score against sociodemographic, COVID-19-related, and disease-related factors and outcomes. The associations between predictor-independent variables in the multivariate analysis and outcome variables was expressed as exponentiated beta coefficients (risk rates), with 95% confidence intervals. Data analysis was performed using SPSS (version 21; IBM Corp., Armonk, NY, USA), with a statistical significance level of α = 0.050.

## 3. Results

The sociodemographic characteristics of the 1024 participants are summarized in Table 1. The sample had a mean age of 33.25 ± 12.43 years, with 52% men. Most participants (56.7%) held university degrees, and over 90% reported medium or higher socioeconomic status. The mean BMI was 26.41 ± 5.16 kg/m^2^. Most were non-smokers (75.1%), and 51.4% reported no chronic diseases.

The acute phase of COVID-19 infection among participants is summarized in Table 2. Most participants (75.9%) had RT-PCR-confirmed COVID-19 infection once, while 24.1% experienced two or more infections. The majority (90.7%) had received three vaccine doses. Only 9.6% presented with pneumonia, and 63.3% managed their illness at home, with just 4.1% requiring hospital admission. The median number of acute symptoms was six (IQR 6), with fever (75.1%) and cough (74%) being the most common. Additional symptoms are detailed in Table 2.

**Figure 1:**
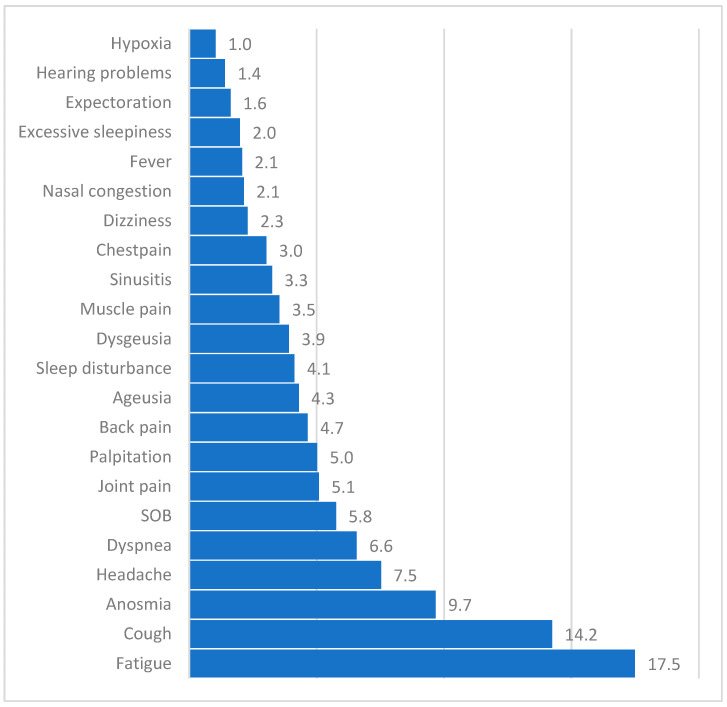
Proportion of most commonly reported long COVID (LC) symptoms (n = 1024). Note: Hypoxia refers to medically confirmed events, not self-diagnosed, and involves SpO_2_ assessment by healthcare providers; dyspnea and shortness of breath (SOB) are listed separately to reflect patient-reported language and capture distinct symptom expressions.

In total, 63.8% of participants reported at least one LC symptom. The most common symptoms included fatigue (17.5%), cough (14.2%), anosmia (9.7%), headache (7.5%), and dyspnea (6.6%). Other less frequent symptoms included shortness of breath, joint pain, palpitations, back pain, and sleep disturbances. Additional symptoms are summarized in Figure 1.

Table 3 presents a descriptive analysis of participants’ perceptions of HRQoL and its subscale scores, along with a bivariate comparison of these perceptions between participants with and without LC. The overall mean (±SD) EQ-5D-5L index score was 0.947 ± 0.11, indicating a high perceived HRQoL among participants. However, a two-sample *t*-test showed that participants without LC reported a significantly higher mean HRQoL score (0.98) than those with LC (0.93) (*p* < 0.001). This pattern of significantly higher averages of reported HRQoL among participants without LC was evident across all EQ-5D-5L index subcomponents (*p* < 0.001), including self-rated ability of mobility, self-care, activities of daily living, comfort level, anxiety/depression, and psychological well-being. Lastly, the overall EQ VAS scale score had a mean of 88.78 ± 13.8 out of 100 points. Participants with LC had a significantly lower EQ VAS score (87.04) compared to those without LC (91.84) (*p* < 0.001).

The multivariable gamma regression analysis (Table 4) assessed factors influencing the mean perceived HRQoL (EQ-5D-5L index score) among COVID-19 survivors. Participants’ sex, body mass index, marital state, and socioeconomic level did not significantly affect HRQoL. However, age had a negative association; for each additional year, HRQoL declined by 0.2% (*p* < 0.001). Pneumonia during acute illness was linked to a 4.4% lower HRQoL (*p* = 0.015). General health self-rating scores positively correlated with HRQoL, with each point increase in EQ VAS score associated with a 3.1% rise in HRQoL (*p* < 0.001). The number of acute COVID symptoms had no significant impact (*p* = 0.125), but each additional LC symptom reduced HRQoL by 0.9% (*p* < 0.001). Depression showed no significant association with HRQoL (*p* = 0.052), while anxiety disorder significantly reduced HRQoL by 13.8% (*p* < 0.001). Similarly, hypertension (HTN) was associated with a 4.9% lower HRQoL (*p* = 0.033). A history of inflammatory bowel disease (IBD) and other measured variables showed no significant associations.

## 4. Discussion

The spread of COVID-19 has impacted societal health and well-being in various ways, with some individuals experiencing symptoms that extend beyond the average duration, known as LC, further deteriorating their HRQoL. This community-based study in the Eastern Province of Saudi Arabia aimed to investigate the impact of COVID-19 and LC on HRQoL. The EQ VAS scale, the number of LC symptoms, age, pre-existing chronic diseases such as HTN and anxiety, and pneumonia during acute presentation strongly influenced the decrease in the mean index score in our sample.

### 4.1. EQ-5D-5L

In our study population, the EQ-5D-5L tool was used to assess HRQoL among COVID-19 survivors. The findings indicate a decline in HRQoL across all participants. Furthermore, differences were observed between individuals who experienced LC and those who did not, suggesting that LC may have a greater impact on HRQoL. These results highlight the importance of long-term monitoring and support for COVID-19 survivors, particularly those affected by persistent symptoms. This finding corroborates the significant positive association observed in the regression model between self-perceived health and predicted HRQoL, where each additional point in the EQ-VAS score was associated with a 3.1% increase in average HRQoL.

These findings are consistent with those of other studies. For instance, a study conducted in Ethiopia reported mean HRQoL and EQ VAS scores of 0.940 and 87, respectively [23]. In India, Barani et al. reported similar scores of 0.925 and 90.68, respectively [17], whereas in China, Ping et al. reported scores of 0.949 and 85.52, respectively [24]. Our results were relatively higher than those reported in the literature. An Italian prospective study conducted among 137 patients with COVID-19, two years post admission, showed a greater decline in mean HRQoL score compared to their pre-infection baseline (from 0.97 to 0.79), with an EQ VAS score of 72.38 [25]. A Vietnamese study also reported lower HRQoL and EQ VAS scores of 0.86 and 78.6, respectively [26]. A quantitative descriptive survey conducted among 200 patients with COVID-19 in Kozhikode reported an average HRQoL score of 0.839 and an EQ VAS score of 72.23 [27]. In European countries, the mean HRQoL and EQ VAS scores varied. For example, while the mean index in Denmark was lower (0.82), the mean score in France matched our results (0.94). The EQ VAS scores in the United Kingdom (70.1) and Portugal (78.4) were significantly lower than that observed in our study [28].

Our mean index and EQ VAS scores were higher than most of the scores reported in the literature. This may be attributed to sociodemographic factors, which likely reflect the generally good health status of our sample. The mean age of 33 years was lower than most studies, and most participants were well educated, of medium-to-high socioeconomic status, and free of chronic conditions. Our study primarily included patients with mild-to-moderate COVID-19, whereas others often examined hospitalized cases. While socioeconomic factors have been linked to low HRQoL in previous research [23,29], this was not evident in our sample.

By the time of the interviews, 90.7% of participants had already received three vaccine doses. The mild impact of COVID-19 on HRQoL among the Saudi population might be attributed to the remarkable efforts of the Saudi Ministry of Health. Since the beginning of the pandemic, comprehensive preventative measures and protocols have been established to limit the spread of infections and maintain daily life routine. For instance, immunization campaigns were organized across all regions of Saudi Arabia, ensuring that citizens received vaccines at no cost. These efforts helped alleviate financial and psychological burdens on families, enhancing HRQoL [30].

All five dimensions of the EQ-5D-5L tool were significantly affected among COVID-19 survivors, with less impairment observed in those without LC. Pain/discomfort and anxiety/depression were the most affected dimensions, which is consistent with the literature [17,23,24,25,26]. Consistently with a recent meta-analysis, 41.5% and 37.5% of patients with LC had a significant decline in pain/discomfort and anxiety/depression dimensions, respectively [31]. A local study reported similar low HRQoL in infected individuals [32]. Another study also reported similar percentages for the same dimensions; nevertheless, individuals with LC had significant impairments in all dimensions of their HRQoL [33]. This may be attributed to the social burden of the pandemic, including distancing protocols that hinder societal engagement [34], and the lack of support systems, exacerbating mental health outcomes [35].

### 4.2. Number of LC Symptoms

The multivariable gamma regression analysis revealed a negative association between the number of LC symptoms and poor HRQoL, with a decrease of 0.9% in HRQoL for each additional LC symptom. These findings are consistent with a longitudinal cohort study conducted in Belgium, which highlighted the impeding impact of the increasing number of LC symptoms on HRQoL; patients with LC reported a mean HRQoL score of 0.65 compared to 0.95 for those without LC [33]. In addition to the quantity, duration, and frequency of each symptom having a tangible influence on overall HRQoL, Samper-Pardo et al. concluded that it is the chronicity of a few symptoms that might cause worse mental sequelae rather than their greatly fluctuating frequency [36], emphasizing the role of emotional and mental well-being [37]. This study is consistent with similar studies stating that LC is significantly associated with poor HRQoL [18,36,38,39,40]. Notably, the present study is among the few to explicitly link the number of LC symptoms with declining mean HRQoL scores. Interestingly, the number of acute COVID-19 symptoms did not influence mean HRQoL scores in the present study.

### 4.3. Age and Chronic Conditions

A fragile immune system, coupled with increasing age and coexisting chronic conditions, contributes to the susceptibility of the elderly to developing LC, which in turn diminishes the overall HRQoL [41,42,43]. In our sample, for each additional year of mean age, the perceived HRQoL declined by 0.2%. Our findings are consistent with previous studies that reported the worsening of HRQoL among individuals aged >60 years in most EQ-5D-5L dimensions, with mean HRQoL scores ranging from 0.554 to 0.889 and EQ VAS scores varying between 50 and 83.84 [11,23,25,26,44].

Hypertension emerged as a precipitating factor among our participants, contributing to a 4.9% reduction in HRQoL. These results are consistent with recently published data [25]. This could be due to multiple reasons. First, the established systemic complications associated with HTN, along with high mortality rates and the psychological burden of the disease [45], can aggravate disease severity and delay recovery from COVID-19 [46]. Second, access to medical consultations and health education was often limited due to quarantine policies or concerns regarding the pandemic [47,48]. Nevertheless, studies that link pre-existing HTN to a decline in HRQoL among patients with LC are scarce and warrant further investigation.

Enrolled individuals with pre-pandemic anxiety disorder reported a mean index score that was 13.8% times lower than the average. This decline may be correlated with the psychological sequelae induced by the COVID-19 pandemic, which is a strong risk factor for initiating or complicating pre-existing mental condition, including anxiety [33]. A similar study reported that patients with pre-existing mental conditions were prone to persistent symptoms [36]. A recent local study demonstrated higher levels of anxiety among infected individuals than among non-infected individuals [32]. This highlights the need for a holistic, multifaceted approach to mental health in individuals with LC. No significant association was observed between low mean HRQoL scores and IBD in our sample. Rosa et al. [49] evaluated the burden of IBD on HRQoL during the pandemic, revealing that disease activity had a more significant impact on HRQoL than pandemic-related concerns. Nevertheless, Nishida et al. [50] highlighted that social restrictions and psychological distress could exacerbate disease activity by deterring medication compliance and access to healthcare for patients with IBD. Studies examining the link between IBD and declining HRQoL among COVID-19 survivors are limited and warrant further investigation.

### 4.4. Pneumonia on Acute Presentation

The intense inflammatory process during acute COVID-19 plays a pivotal role in immune dysregulation [51]. Thus, the severity of COVID-19 can negatively influence the HRQoL and subsequently contribute to the development of LC [52]. In our sample, patients who had pneumonia on acute presentation had a mean HRQoL index score that was 4.4% lower than that of individuals with mild symptoms. Few studies have found a significant link between the severity of COVID-19 and persistence of symptoms and poor quality of life [40,53]. However, Garrigues et al. [54] reported that admission to either the ward or ICU did not affect the development of LC or poor HRQoL. Nonetheless, the impact of severe acute presentation of COVID-19 remains unclear and warrants further investigation.

### 4.5. Strengths and Limitations

This study is one of the largest cross-sectional surveys of LC in the Middle East. It achieved a diverse representation across key sociodemographic variables, including sex, marital status, and education, strengthening the representativeness and generalizability of the findings. However, the study has some limitations. It relies on patients’ memory to recall events, introducing potential recall bias. Additionally, individuals with LC symptoms may have been more inclined to participate, possibly leading to selection bias. Nonetheless, the sample was balanced regarding chronic disease status, with 51.4% of participants reporting no chronic diseases and 48.6% having one or more, minimizing the potential impact of selection bias. Finally, the cross-sectional design does not allow for the establishment of causality.

## 5. Conclusions

This study adds to the epidemiological evidence and has highlighted the multisystemic impact of COVID-19 on HRQoL and the potential deterioration associated with symptoms beyond the active infection phase. Factors such as age, the number of LC symptoms, pneumonia at acute presentation, chronic diseases such as HTN, and anxiety were significant contributors to the lower HRQoL scores in our study. These factors could serve as useful predictors of a poor quality of life. Therefore, multidisciplinary efforts are imperative, and implementing effective interventions, including rehabilitation and exercise programmes, could help prevent further deterioration and mitigate additional physical, psychosocial, and economic setbacks.

## Figures and Tables

**Table 1:** Descriptive analysis of people’s sociodemographic characteristics (n = 1024).

Characteristic	Mean (SD) or n (%)
Sex	
Female	492 (48%)
Male	532 (52%)
Age (years), mean (SD)	33.25 (12.43)
Marital status	
Never married	464 (45.3%)
Married	560 (54.7%)
Level of education	
High school education or less	319 (31.2%)
University degree or diploma	665 (64.9%)
Higher studies	40 (3.9%)
Socioeconomic status level	
Very low	4 (0.4%)
Low	54 (5.3%)
Medium	632 (61.7%)
High	177 (17.3%)
Very high	157 (15.3%)
BMI (kg/m^2^), mean (SD)	26.41 (5.16)
Smoking	
Never smoked	769 (75.1%)
Former smoker	74 (7.2%)
Current smoker	181 (17.7%)
Chronic disease	
None	526 (51.4%)
One	296 (28.9%)
Two or more	202 (19.7%)

Note: BMI: body mass index; SD: standard deviation.

**Table 2:** Descriptive analysis of people’s acute phase of COVID-19 infection (n = 1024).

Characteristic	Median (IQR) or n (%)
How many times did you get PCR-confirmed COVID-19 infection?	
Once	777 (75.9%)
Twice	201 (19.6%)
Three times	40 (3.9%)
Four times	2 (0.2%)
Five times	4 (0.4%)
How many COVID-19 vaccine shots did you receive?	
None	6 (0.6%)
One dose	2 (0.2%)
Two doses	76 (7.4%)
Three doses	929 (90.7%)
Four doses	11 (1.1%)
Presented to the hospital with pneumonia	
No	926 (90.4%)
Yes	98 (9.6%)
Need for healthcare	
No need/Asymptomatic	175 (17.1%)
Home management	648 (63.3%)
ER/Out-patient services	159 (15.5%)
Required hospital admission	42 (4.1%)
Hospital admission type	
No admission	982 (95.9%)
Hospital floor admission	32 (3.1%)
ICU admission	10 (1%)
Acute COVID-19 symptoms, median (IQR)	6 (6)
Experienced long COVID symptoms	
No	371 (36.2%)
Yes	653 (63.8%)
Acute COVID-19 symptoms	
Fever	722 (75.1%)
Cough	712 (74%)
Fatigue	495 (51.5%)
Headache	410 (42.6%)
Anosmia	397 (41.3%)
Sore throat	375 (39%)
SOB	370 (38.5%)
Runny nose	351 (36.5%)
Ageusia	294 (30.6%)
Nasal congestion	283 (29.4%)
Joint pain	269 (28%)
Back pain	262 (27.2%)
Chest pain	247 (25.7%)
Chills	244 (25.4%)
Muscle pain	237 (24.6%)
Appetite	229 (23.8%)

Note: SD: standard deviation; IQR: interquartile range; SOB: shortness of breath.

**Table 3:** Descriptive analysis of quality of life (QoL) indicators among participants with and without long COVID (LC) (n = 1024).

	Mean (SD)			
	Overall	No LC (n = 371)	LC (n = 653)	*p*-Value ^a^
EQ-5D-5L Index Score	0.947 (0.111)	0.98 (0.08)	0.93 (0.12)	<0.001
Self-Rated Mobility Ability	4.84 (0.53)	4.93 (0.37)	4.78 (0.59)	<0.001
Self-Rated Self-Care Ability	4.93 (0.33)	4.97 (0.24)	4.91 (0.70)	<0.001
Self-Rated Ability to Perform ADL	4.84 (0.50)	4.94 (0.35)	4.80 (0.55)	<0.001
Comfort Level	4.74 (0.61)	4.91 (0.40)	4.65 (0.69)	<0.001
Anxiety/Depression (Psychological WB)	4.73 (0.66)	4.86 (0.46)	4.65 (0.74)	<0.001
EQ VAS Scale (Out of 100)	88.78 (13.8)	91.84 (11.78)	87.04 (14.58)	<0.001

Note: ^a^ two-sample *t*-test; ADL: activities of daily living, WB: well-being, EQ VAS: EuroQol Visual Analogue Scale.

**Table 4:** Multivariable gamma regression analysis of health-related quality of life (HRQoL) scores among COVID-19 survivors (n = 1024).

Variable	Adjusted Risk Rate (RR)	95% CI	*p*-Value
Number of LC symptoms	0.991	(0.99, 1)	<0.001
Gender			
Male	Reference		
Female	1.007	(0.98, 1.03)	0.602
Age (Years)	0.998	(0.996, 0.999)	<0.001
Marital Status			
Never married	Reference		
Married	1.021	(0.99, 1.05)	0.137
Socioeconomic Status			
Very low–Low	Reference		
Medium–High	1.008	(1, 1.02)	0.207
BMI (kg/m^2^)	0.999	(0.997, 1.001)	0.379
Number of Acute COVID Symptoms at Presentation	1.007	(1, 1.02)	0.125
Pneumonia at Presentation	0.956	(0.92, 0.99)	0.015
EQ VAS Scale	1.031	(1.02, 1.04)	<0.001
History of Hypertension	0.951	(0.91, 1)	0.033
History of Depression	0.931	(0.87, 1)	0.052
History of Anxiety Disorder	0.862	(0.81, 0.92)	<0.001
History of Inflammatory Bowel Disease	0.940	(0.88, 1.01)	0.076
(Intercept)	0.937	(0.86, 1.02)	0.119

Note: BMI: body mass index. Outcome: overall mean of health-related quality of life index score (EQ-5D-5L).

## Data Availability

The data used in this study are not publicly available due to privacy concerns and institutional regulations. However, de-identified data supporting the findings of this study may be available from the corresponding author upon reasonable request, provided it complies with institutional data sharing policies and privacy requirements.

## Abbreviations

The following abbreviations are used in this manuscript:

COVID-19	Coronavirus disease 2019
LC	Long COVID
HRQoL	Health-related quality of life
SARS-CoV-2	Severe acute respiratory syndrome coronavirus 2
EQ-5D-5L	EuroQoL 5 Dimensional 5 Levels
RT-PCR	Reverse transcription polymerase chain reaction
EQ VAS	EuroQol Visual Analogue Scale
SD	Standard deviation
IQR	Interquartile range
HTN	Hypertension
IBD	Inflammatory bowel disease
BMI	Body mass index
SOB	Shortness of breath
ADL	Activities of daily living
WB	Well-Being
